# Effect of Adsorption Deacidification on the Quality of Peony Seed Oil

**DOI:** 10.3390/foods12020240

**Published:** 2023-01-05

**Authors:** Zhi Wang, Xuan Ma, Chang Zheng, Weijun Wang, Changsheng Liu

**Affiliations:** Hubei Key Laboratory of Lipid Chemistry and Nutrition, Key Laboratory of Oilseeds Processing, Oil Crops and Lipids Process Technology National & Local Joint Engineering Laboratory, Oil Crops Research Institute of the Chinese Academy of Agricultural Sciences, Ministry of Agriculture, Wuhan 430062, China

**Keywords:** peony seed oil, deacidification, bioactive compounds, alkali microcrystalline cellulose

## Abstract

To overcome the issues in the traditional deacidification processes of peony seed oil (PSO), such as losses of neutral oil and trace nutrients, waste discharge, and high energy consumption, adsorption deacidification was developed. The acid removal capacity of adsorbent-alkali microcrystalline cellulose was evaluated using the isothermal adsorption equilibrium and the pseudo-first-order rate equation. The optimized adsorption deacidification conditions included adsorbent-alkali microcrystalline cellulose at 3%, a heating temperature of 50 °C, and a holding time of 60 min. The physicochemical, bioactive properties, antioxidant capacities, and oxidative stabilities of PSO processed by alkali refining and oil-hexane miscella deacidification were compared under the same operating conditions. Fatty acid content was not significantly different across all three methods. The deacidification rates were 88.29%, 98.11%, and 97.76%, respectively, for adsorption deacidification, alkali refining, and oil-hexane miscella deacidification. Among the three deacidification samples, adsorption deacidification showed the highest retention of tocopherols (92.66%), phytosterols (91.96%), and polyphenols (70.64%). Additionally, the obtained extract preserved about 67.32% of the total antioxidant activity. The oil stability index was increased 1.35 times by adsorption deacidification. Overall, adsorption deacidification can be considered a promising extraction technology in terms of quality as compared to alkali refining and oil-hexane miscella deacidification.

## 1. Introduction

The peony (*Paeonia Suffruticosa*) is a kind of woody oleaginous plant and industrial crop which has broad development prospects, and its cultivation area is increasing annually [1]. Currently, the planting area is more than 3.2 million mu, and the annual output of peony seeds is about five hundred thousand tons in China. Tree peony has been widely cultivated for the high rate of oil yield from its seeds (24.12–37.83%) [2]. Peony seed oil (PSO) is notable for containing abundant unsaturated fatty acids (greater than 90%) and an α-linolenic acid content greater than 40% [2,3,4]. PSO is rich in bioactive substances such as phytosterols, tocopherols, and polyphenolic compounds [5,6,7]. These beneficial components play an important role in producing health-promoting effects [8]. However, crude oil has the characteristic of high acid value [9], which can alter the taste and affect the quality of edible oils by accelerating the oxidative rancidity [10,11]. Therefore, the deacidification process needs to be employed. At present, alkali refining, molecular distillation, liquid–liquid extraction, enzymatic esterification, and adsorption refining have been widely used in the deacidification of vegetable oils [12]. Among them, alkali refining for deacidification is economical and suitable for the separation of oil and soap. However, it can cause too great a loss of neutral oil and active ingredients, resulting in a large amount of wastewater [13,14,15]. Molecular distillation has little loss during refining but requires high operating temperatures, and high phosphorus content in the oil, and high energy consumption [16]. The deacidification of oils by liquid–liquid extraction has been studied more extensively. It has low energy consumption, mild reaction conditions, and high retention of nutrients. However, it generates a large amount of solvent, causing environmental pollution problems [17,18]. As a result, ionic liquids with green solvents have been developed [19]. Enzymatic esterification deacidification has been a hot research topic. It is a milder and greener process, and the beneficial micronutrients are not destroyed during processing. It produces high oil yield, but catalysts and prices are important factors restricting development [20,21]. In comparison, the adsorption technique appears to be a good choice owing to its mild conditions, simplicity of operation, and low cost. A wide variety of absorbents such as calcium carbonate, nano-silica, alumina, activated carbon, modified cellulose, chitosan, and ion exchange resin have been implemented for the deacidification of edible oils [22,23,24].

Microcrystalline cellulose can be produced from the partial hydrolysis of cellulose with diluted mineral acids, and has the characteristics of a low degree of polymerization, large specific surface area, and good adsorption. Microcrystalline cellulose has been widely used and applied as a potential bio-additive in diverse applications, including water treatment, cosmetics, and the pharmaceutical and food industries [25,26]. Some studies have reported that alkali treatment can destroy the crystalline area of cellulose and increase the porosity of the feedstock while improving the chemical reactivity [27]. Therefore, alkali microcrystalline cellulose deacidification efficiencies were evaluated in this paper. The influences of temperature, additional amounts, time, and adsorption equilibrium on the adsorbent-alkali microcrystalline cellulose’s effect were also considered.

In the present work, we studied the influence of the adsorption deacidification processing on the acid removal rate, the fatty acid content, the amount of multiple bioactive compounds, the antioxidant capacity, and the transferred amount of acid during the deacidification process of PSO. The main goal was to maximize the transfer of acid and minimize the losses of neutral oil and nutraceutical compounds. Adsorption deacidification technology is an economical and environmentally friendly refining technology because it has mild reaction conditions and zero wastewater is produced during the production process. It provides a new strategy and shows great potential in the deacidification of oils.

## 2. Materials and Methods

### 2.1. Chemicals and Reagents

5α-cholestane, 37 standard fatty acid methyl ester mixtures, α-tocopherol, γ-tocopherol, δ-tocopherol, fucosterol, standard polyphenol extract, BSTFA + TMCS, campesterol, squalene, 24-Methylenecycloartan-3β-ol, β-sitosterol, 2,2-diphenyl-1-picrylhydrazyl (DPPH), 2,4,6-tris(2-pyridyl)-s-triazine (TPTZ), and 6-hydroxy-2,5,7,8-tetramethylchroman-2-carboxylic acid (Trolox) were obtained from Sigma-Aldrich (Saint Louis, MO, USA). All standards had purity ≥ 97%. Folin–Ciocalteu phenol reagent was purchased from Shanghaiyuanye Bio-Technology Co., Ltd. (Shanghai, China). Methanol (LC-MS grade) was purchased from Fisher, USA. Acetonitrile (LC-MS grade) was purchased from Merck KGaA (Darmstadt, Germany). Alkaline microcrystalline cellulose, and other chemicals and reagents of analytical grade, were obtained from Sinopharm Chemical Reagent Co., Ltd. (Beijing, China). Silica dioxide and activated clay were purchased from cNPC Konecranes Technology Co., Ltd. (Wuhan City, Hubei Province, China). All the reagents were directly used without further purification.

### 2.2. Plant Materials

Fresh tree peony seeds (*P*. Austin) used in this study were purchased from Heze (Heze City, Shandong Province, China). The moisture content was 7.11%, and the acid value was 5.81 (KOH mg/g). The processed PSO was pressed with a cold-press machine (CA59, Moenchengladbach, Germany), and centrifuged at 10,000 rpm for 20 min (Sorvall Stratos, Thermo Fisher Co., Ltd., Waltham, MA, USA). All samples were sealed in amber glass vials and stored in a refrigerator (4 ± 2 °C).

### 2.3. Preparation of Alkaline Microcrystalline Cellulose

We prepared a sodium hydroxide solution with a mass fraction of 18% (24 °Be) and mixed the microcrystalline cellulose powder and sodium hydroxide solution at a ratio of 1:10. This was stirred and allowed to react at 30 °C for 30 min, then centrifuged to obtain a precipitate and rinsed with 95% ethanol. It was then placed in a 60 °C oven to dry, and the dried material was pulverized to 60 meshes to obtain adsorbent-alkali microcrystalline cellulose. It was then stored in a sealed and dry condition for later use.

#### 2.3.1. Adsorption Equilibrium Experiment of Alkaline Microcrystalline Cellulose

Using PSO and oleic acid as raw materials, six kinds of PSO samples were prepared, and their initial acid values were 1.97 (KOH mg/g), 3.78 (KOH mg/g), 5.74 (KOH mg/g), 7.27 (KOH mg/g), 8.76 (KOH mg/g), 10.03 (KOH mg/g), and 11.97 (KOH mg/g). Then, 3% adsorbent-alkali microcrystalline cellulose was added and the equilibrium adsorption time was 1 h. The acid value of PSO after adsorption and deacidification was measured, and then different acid values were obtained. The adsorption isotherms were mainly obtained from the Langmuir adsorption isotherm equation for adsorption rate and adsorption capacity:

(1) $$\;\mathrm{Adsorption}\;\mathrm{rate}=(1-\frac{{\mathrm{AV}}_{1}}{{\mathrm{AV}}_{0}})\times 100\;\%\;$$

where AV_1_ is the acid value after adsorption (mg/g) and AV_0_ is the initial value (mg/g).

The adsorption capacity calculation formula is given by:

(2) $${\;Q=(\mathrm{AV}}_{0}-{\mathrm{AV}}_{1})\times \frac{282}{56.1}\times \frac{M_{\mathrm{oil}}}{m}\;\;\;\;\;\;\;\;\;\;\;$$

where Q is the adsorption capacity (mg/g); M_oil_ is the mass of PSO (g); and m is the additive amount of adsorbent (g).

The Langmuir adsorption isotherm equation according to Mubarak et al. [28]:

(3) $$\;\;\;\frac{\mathrm{AV}}{Q}=\frac{\;\mathrm{AV}}{Q_{e}}+\frac{1}{{\mathrm{KQ}}_{e}}\;\;\;$$

where AV is the acid value of PSO before adsorption equilibrium (mg/g); Q_e_ is the equilibrium adsorption capacity (acid value of PSO at adsorption equilibrium) (mg/g); and K is the Langmuir adsorption constant (g/mg).

#### 2.3.2. Alkaline Cellulose Adsorption Kinetic Test

The acid value is converted into free fatty acid (FFA) (calculated as oleic acid), and the formula is as follows:

(4) $$\mathrm{FFA}\;(\mathrm{oiled}\;\mathrm{acid})=\frac{\left(V-V_{0}\right)\;\times \;282\;\times \;C}{\mathrm{Moil}}$$

The amount of oleic acid adsorbed by alkaline microcrystalline cellulose can be known from the following calculation formula:

(5) $$Qt=\frac{\mathrm{AV}}{Q_{e}}+\frac{1}{{\mathrm{KQ}}_{e}}$$

### 2.4. Preparation of Samples

#### 2.4.1. Adsorption Refining

The samples were prepared by adding 1% (*w*/*w*) of silicon dioxide powder to 200 g crude PSO. The samples were then kept in a water bath at 40 ± 1 °C and stirred at the rate of 150 rpm using a magnetic stirrer for 20 min. They were subsequently centrifuged at 4000 rpm for 10 min and then filtered. Then, 2.5% alkaline microcrystalline cellulose was added and allowed to adsorb with magnetic stirring at 45 °C for 1 h.

#### 2.4.2. Chemical Refining

The oil sample was heated to 80 °C with slow stirring, then 3% oil-weight isothermal distilled water and 0.1% citric acid were added. Then, speed was adjusted appropriately, and then reduced after stirring for 20 min to promote colloidal flocculation. Stirring was stopped when the colloid and oil were clearly separated, then the sample was centrifuged at 5000 r/min for 20 min, and set aside.

The oil sample was subsequently heated to 30 °C under stirring, and the amount of alkali added was calculated according to Wang et al. [18]. According to the acid value of the oil sample, we slowly added 18 °Be NaOH, stirring quickly for 20 min (150 rpm), then slowly while the sample was heated to 60 °C at a rate of 1 °C/min. The sample was then stirred for 10 more minutes to promote the flocculation of the colloid. After the soap and oil were separated, stirring was stopped and the sample was centrifuged at 5000 r/min for 20 min and set aside.

#### 2.4.3. Hexane Miscella Refining

PSO-hexane miscella with 50 g/100 g was heated to a certain temperature (60 °C) in a three-neck round bottom flask and mixed using a magnetic stirrer at a stirring speed of 150 rpm. After being heated to reaction temperature, certain amounts of different alkali solutions were added slowly and the reaction was initiated. In all refining methods, reaction mixtures were centrifuged at 5000 r/min for 20 min after the reaction was finished and then set aside. All operations and assays were carried out were conducted in triplicate.

### 2.5. Physicochemical Properties of PSO

The acid value (AV) was measured according to GB 5009.229-2016 [29]. Briefly, the acid value is determined through titration method. The oil sample was fully dissolved in an organic solvent, and then titrated with KOH standard solution. Finally, the titration end point was defined by the color reaction of the indicator. The acid value was expressed as the milligrams of potassium hydroxide (KOH) required neutralizing 1 g of free fatty acid.

### 2.6. Determination of the Profile of Fatty Acids

Determination of the fatty acids content was conducted according to GB5009.168-2016 [29]. Briefly, we accurately weighed 80 mg PSO, added 0.5 mL 0.5 M sodium methoxide and 2.5 mL n-hexane, and then the supernatant was injected into a GC (GC7890A, Agilent, Santa Clara, CA, USA) equipped with a flame ionization detector (FID) for quantifying fatty acid methyl esters. The column was DB-FFAP (30 m × 250 μm id × 0.25 μm thickness). The temperatures of the injector and detector were both set at 300 °C, and the flow rate of nitrogen was 1.5 mL/min with a split ratio of 80:1. The column temperature was held for 9 min at 210 °C, then it was increased by 20 °C/min to 250 °C and maintained for 10 min. Its content was calculated according to the external standard curve and included palmitic acid (y = 455.05x − 63.569, R^2^ = 0.99), stearic acid (y = 351.9x − 13.78, R^2^ = 0.99), oleic acid (y = 363x − 78.457, R^2^ = 0.99), linoleic acid (y = 348.69x − 1.7031, R^2^ = 0.99), and linolenic acid (y = 347.39x − 11.765, R^2^ = 0.99).

### 2.7. Determination of the Bioactive Compounds

The tocopherol, phytosterols, and squalene were measured according to Wang et al. [9].

### 2.8. Phenolic Compounds Extraction

Mixed-mode SPE cartridges were sequentially preconditioned with 5 mL methanol and 5 mL n-hexane each. An equivalent of 1.00 ± 0.01 g of oil sample was exactly weighted and diluted to 5 mL with n-hexane by 5 min vortex. The oil sample was loaded on the SPE cartridges at a flow rate of 1.0 mL min^−1^, and washed with 3 mL of ethyl acetate/n-hexane (*v*/*v*, 1:9). Then, the analytes were eluted into the centrifuge tube with 3 × 1.5 mL of 0.1% formic acid in methanol. The eluent was evaporated to dryness with nitrogen at 40 °C and reconstituted with 200 µL of methanol solution.

### 2.9. UPLC Quantified the Phenolic Compounds

The phenolic compounds were identified and quantified via ultraperformance liquid chromatography (UPLC) using an Acquity system (Waters, Milford, MA, USA). The UPLC analysis was conducted on a Waters Acquity BEH T3 C18 column (2.1 × 100 mm, 1.7 μm, Waters, Milford, MA, USA). Pure acetonitrile was used as mobile phase A and a 0.1% formic acid (Merck, Darmstadt, Germany) solution was used as mobile phase B with a flow rate of 0.3 mL/min. The following were the gradient elution conditions: the starting conditions were 16% A, 84% B, 24% A at 5 min; 55% A at 7 min; 65% A at 8 min; 98% A at 10 min; 98% A at 15 min; and 16% A at 18 min. The injection volume of each sample was 3 μL, while the temperature of the sample and column were 30 °C and 25 °C, respectively.

## 3. Determination of the Oxidative Stabilities of the Oils

The total phenolic content (TPC) was determined through the Folin–Ciocalteu colorimetric method [30] with some modifications. The results are expressed in gallic acid equivalents (mg GAE/100 g). The ferric-reducing antioxidant power (FRAP) and 2,2-diphenyl-1-picrylhydrazyl (DPPH) were determined using a previously described method. [30]. Trolox was tested to establish a standard curve. The results of DPPH and FRAP are expressed as micromoles of Trolox equivalents per 100 g of sample (μmol TE/100 g). The induction period (IP) was determined according to Azadmard et al. [31].

The results are expressed as mmol.

(6) $$\mathrm{Trolox}\;\mathrm{equivalents}\;100\;g^{-1}\;\mathrm{DW}.$$

### Statistical Analysis

Mean values of each sample were obtained from three replications and used for further analysis, and the experiment data are expressed as the value (mean ± SD) of the average of the triplicate test. Statistical analysis was performed using SPSS 20.0 (SPSS Inc., Chicago, IL, USA). The data were statistically evaluated by variance analysis (ANOVA) followed by Duncan’s test at a significance level of *p* < 0.05. A principal component analysis (PCA) model was generated to overview the relationship of samples. Origin 9.0 software (Origin Lab Corp., Northampton, England) was used to drawn the figures.

## 4. Results

### 4.1. Effects of Different Deacidification Conditions on Acid Value

**Figure 1:**
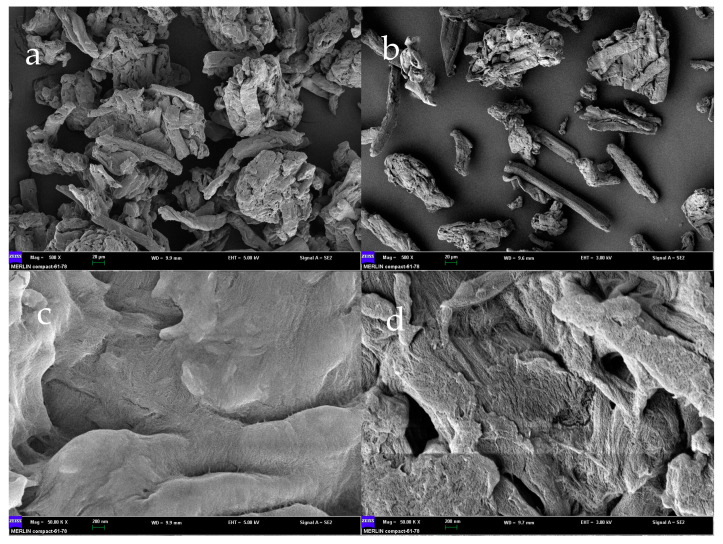
SEM of alkaline microcrystalline cellulose ((**a**): 20 µm and (**c**): 200 nm) and microcrystalline cellulose ((**b**): 20 µm and (**d**): 200 nm). SEM: scanning electron microscopy.

The deacidified adsorbent-alkali microcrystalline cellulose was prepared with 12% NaOH. The surface morphology of the prepared alkaline microcrystalline cellulose was observed using scanning electron microscopy (SEM) at an accelerating voltage of 10.0 kV with a figure size of 20 µm and 200 nm. As can be seen in Figure 1b,d, the results of SEM before and after alkalinization of microcrystalline cellulose showed that the surface of microcrystalline cellulose without alkali treatment was smooth, firm, and undamaged, and the connections between structures were looser, which was unfavorable to the adsorption reaction. In Figure 1a,c, we can observe that the reaction of microcrystalline cellulose with alkali resulted in tight junctions between individual microcrystalline cellulose that clumped together to form agglomerates and showed many ordered and clear voids and pits in the prepared SEM. 

The adsorption mechanism was as follows: The cellulose alkali and cellulose-activated hydroxyl on the surface could undergo adsorption and neutralization reaction with FFAs. The cellulose lipid salt was produced, which was connected to the long cellulose chain, and FFA could be removed by separation. This structure was conducive to the adsorption of FFAs. By adding 3% alkaline cellulose, the acid value dropped from 5.81 (KOH mg/g) to 0.68 (KOH mg/g), and the deacidification rate reached 88.29% of the demand for the industry-standard GB/T 40622-2021 (Food industry standard of the People’s Republic of China for PSO). The results were generally consistent with previous studies, but there was some variability in the results from SUN and LU et al. [32,33]. The two alternative deacidification methods, alkali refining and oil-hexane miscella deacidification, could reach deacidification rates of 98.11% and 97.76%, respectively. However, compared with alkali refining deacidification, adsorption deacidification does not require water to wash, which can reduce water and oil consumption, and adsorption deacidification simplifies the process and reduces the cost. The FFAs and a small amount of oil adsorbed by adsorbent-alkali microcrystalline cellulose can be recycled, and adsorbent-alkali microcrystalline cellulose is a cellulosic biomass material, which can be used as fuel. In addition to reducing energy consumption and costs, adsorbent-alkali microcrystalline cellulose is also safe and environmentally friendly. However, the deacidification rates of the alkali refining and oil-hexane miscella deacidification methods were higher than that of adsorption deacidification, which was possibly a result of the adsorption equilibrium process in adsorption deacidification.

### 4.2. Adsorption Equilibrium Effect of Adsorbent-Alkali Microcrystalline Cellulose on Different Acid Values

**Figure 2:**
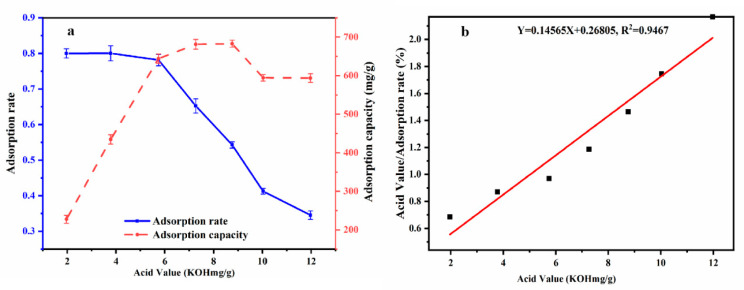
Different acid values on adsorption rate and adsorption capacity (**a**); Adsorption isotherm of acid value at equilibrium concentration Langmuir equation (Xe/q) (**b**).

Based on the above condition, adsorption equilibrium effect experiments were carried out at different acid values (1.97~11.96 (KOH mg/g)) while other variables were set at a reaction temperature of 50 °C for 60 min, adding 3% adsorbent-alkali microcrystalline cellulose. From Figure 2a, we can observe that for the acid values less than 6 (KOH mg/g), the adsorption effect was good, and the absorption rate reached up to 80.03%.

From the relationship curve comparing PSO with different acid values to equilibrium adsorption capacity (Figure 2b), it can be seen that with the continuous increase in acid value, the adsorption capacity also increased, but the adsorption rate decreased. When the original acid value was 5.74 (KOH mg/g), a higher adsorption rate and adsorption capacity could be achieved. Therefore, it was shown that adsorbent-alkali microcrystalline cellulose is suitable for oil with an original acid value of about 6 (KOH mg/g) and can be used for deacidification in this experiment.

The Langmuir adsorption isotherm was similar to the adsorption isotherm in this study. Figure 2b shows the adsorption isotherm based on the Langmuir equation, (Xe/q) and the acid value at equilibrium concentrations (Y = 0.14565X + 0.26805, R^2^ = 0.94). It could be calculated by the straight-line equation, where Qm = 6.8658 (mg/g) and b = 0.5433. The adsorption capacity could reach 6.8658 mg/g, which could effectively remove FFAs in PSO. This shows that the alkaline microcrystalline cellulose prepared by this method has a good adsorption effect. The results are generally consistent with HU [34] in showing that alkaline microcrystalline fibers can effectively remove FFAs from oils and fats and are excellent adsorbent materials for FFA adsorption.

### 4.3. Effects of Adsorption Conditions on Deacidification

**Figure 3:**
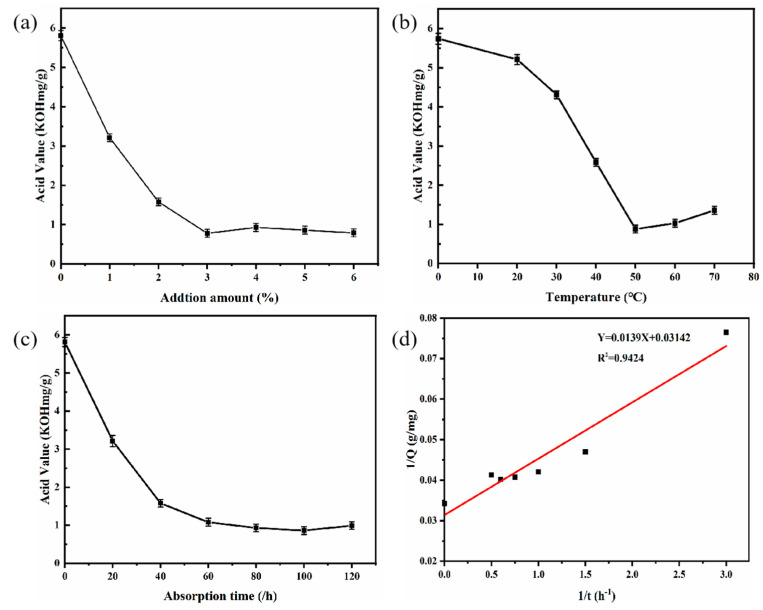
Effects of additional amounts of AMC fiber (**a**); adsorption temperature (**b**); and adsorption time (**c**) on deacidification. And 1/Q; 1/t (**d**) (pseudo-first-order rate model). AMC: Adsorbent-alkali microcrystalline cellulose.

Various amounts of adsorbent-alkali microcrystalline cellulose fibers were used for the deacidification of PSO to study the effect of adsorbent quantity. The reaction was carried out at 50 °C and shaken at 120 r/min for 120 min, and the results are as shown in Figure 3a.

The acid value of the oil sample decreased from 5.81 (KOH mg/g) ± 0.05 to 0.78 ± 0.04 (KOH mg/g) as the amounts of adsorbent-alkali microcrystalline cellulose fibers increased from 0 to 3%, and showed no major change when the amount was further increased to 6%. These results exhibited the feasibility of using adsorbent-alkali microcrystalline cellulose fibers as adsorbents for the deacidification of PSO, and the optimum amount of adsorbents was 3%.

The effect of adsorption temperature on deacidification was investigated using 3% of adsorbent-alkali microcrystalline cellulose. As can be seen in Figure 3b, the acid value of oil samples was decreased from 5.21 ± 0.06 (KOH mg/g) to 0.88 ± 0.05 (KOH mg/g) as the temperature rose from 20 to 50 °C, yet increased when the temperature was further increased the temperature from 50 and 70 °C. Therefore, the optimum adsorption temperature was 50 °C.

The effect of adsorption time on deacidification is shown in Figure 3c. The adsorption deacidification reaction was carried out at 50 °C with 3% adsorbent-alkali microcrystalline cellulose fibers added. The sample was shaken at 120 r/min for the designated time. With the increase in adsorption time from 0 to 60 min, the acid value decreased from 5.81 ± 0.05 (KOH mg/g) to 1.08 ± 0.03 (KOH mg/g), indicating that longer adsorption time leads to better deacidification effects. However, there was not a dramatic reduction in acid value when further lengthening the adsorption time from 60 min to 120 min, similar to the report of Wang et al. [35]. Therefore, the optimum adsorption time was 60 min. To further investigate of the adsorption process, the experimental data were analyzed by various adsorptive models, and the results (Figure 3d) showed that the data fitted well with the pseudo-first-order rate equation (Y = 0.0139X + 0.03142, R^2^ = 0.94)

### 4.4. Fatty Acid Compositions, Tocopherols, and Phytosterols in PSO

The bioactive compounds of PSO produced by various adsorption deacidification methods were investigated and the results of alkali refining and oil-hexane miscella deacidification were compared with the crude oil obtained from untreated seed samples. The fatty acid composition and the tocopherol and phytosterol contents of PSO produced through the three deacidifications methods are presented in Table 1. The main fatty acids of peony seeds found by quantitative detection were palmitic acid (5.18 mg/100 g), stearic acid (1.83 mg/100 g), oleic acid (26.01 mg/100 g), linoleic acid (29.16 mg/100 g), linolenic acid (42.97 mg/100 g), of which saturated fatty acids (SFAs) accounted for 6.66% of the total and unsaturated fatty acids(UFAs) accounted for 93.33% of the total. After deacidification by adsorption deacidification, alkali refining, and oil-hexane miscella deacidification, the fatty acid content decreased to a certain extent, probably due to the FFAs being largely neutralized in this process, but most of the fatty acids showed little change (the rate of change was less than 10%).

The UFA content decreased by 10.27%, 16.48%, and 13.67%, respectively, after adsorption deacidification, alkali refining, and oil-hexane miscella deacidification. The UFA content of the sample that underwent adsorption deacidification decreased the least and was greatly retained in the oil. However, there was no significant difference in the change in UFA content among adsorption deacidification, alkali refining, and oil-hexane miscella deacidification (*p* > 0.05). PSO is a precious vegetable oil resource rich in UFAs (>80%) and with a high proportion of n-3 fatty acids. It has a low ratio of n-6/n-3 ratio (<4.0) which is beneficial to human health, and it can reduce the risk of atherosclerosis, coronary heart disease, and cardiovascular disease [6,36,37].

The main tocopherol in PSO was γ-tocopherol, and the content was 843.12 mg/kg. There were also small amounts of α-tocopherol (47.88 mg/kg) and δ-tocopherol (79.12 mg/kg). After adsorption deacidification, alkali refining, and oil-hexane miscella deacidification, it can be seen that the main reduction occurred in γ-tocopherol, with its content decreasing by 66.67 mg/kg, 132.78 mg/kg, and 121.95 mg/kg, respectively. There was no significant difference between α and δ-tocopherol after deacidification (*p* > 0.05). The retention rates of total tocopherol were 92.66%, 71.85%, and 75.24%, respectively, after adsorption deacidification, alkali refining, and oil-hexane miscella deacidification. The high temperature was the main reason for the loss of tocopherols in the alkaline refining process. Thermal degradation occurred at high temperatures through oxidative or chemical reactions when the oil was exposed to air or heated, such as tocopherols ester formation. Adsorption deacidification preserved its tocopherol content to a greater extent, followed by oil-hexane miscella deacidification. Tocopherols haven been reported to be an active substances with strong antioxidant function, and therefore also show good antioxidant properties in the oil obtained by adsorption deacidification [38].

As can be seen from Table 1, the content of squalene was significantly reduced (*p* < 0.05), from the original 60.61 mg/kg to 9.74 mg/kg, 5.90 mg/kg, and 6.31 mg/kg after treatment with adsorption deacidification, alkali refining, and oil-hexane miscella deacidification, respectively. The retention rates of squalene by adsorption deacidification, alkali refining, and oil-hexane miscella deacidification. were only 16.07%, 9.73%, and 10.40%. The main sterols in PSO were β-sitosterol and fucosterol, the contents of which were 1684.62 mg/kg and 772.53 mg/kg, respectively, followed by cyclopineapple and Δ5-oatasterol. After adsorption deacidification, alkali refining, and oil-hexane miscella deacidification, the total retention rates of sterols were 91.96%, 73.85%, and 76.45%, respectively. In particular, β-sitosterol decreased from its initial value to 1539.51 mg/kg, 1422.18 mg/kg, and 1431 mg/kg, and fucosterol decreased by 6.93%, 38.76%, and 30.91%, respectively. Compared with adsorption deacidification, alkali refining leads to a lower sterol content, because the saponification reaction process is more intense in alkali refining. Free phytosterols can be transferred to soapstock by liquid–liquid distribution and finally removed by centrifugation. Adsorption deacidification is a relatively mild reaction, resulting in better retention of sterols and squalene [18].

### 4.5. Quantitative Analysis of Polyphenol Compounds of PSO

**Figure 4:**
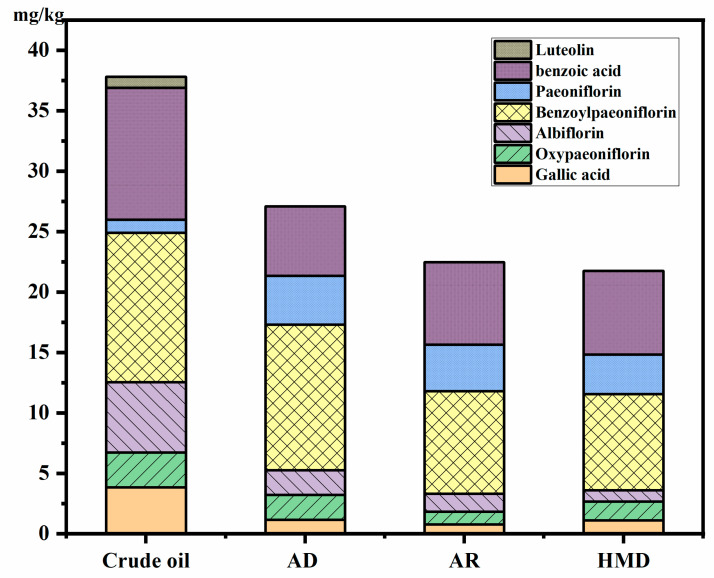
Effects of different deacidification methods on polyphenol components of peony seed oil. AD: adsorption deacidification, AR: alkali refining, HMD: oil-hexane miscella deacidification.

Paeoniflorin (1.08 mg/kg), oxypaeoniflorin (2.90 mg/kg), paeoniflorin (12.34 mg/kg), benzoylpaeoniflorin (5.82 mg/kg), luteolin (0.90 mg/kg), benzoic acid (10.92 mg/kg), and gallic acid (3.83 mg/kg) are the targeted bioactive compounds in PSO, which play essential roles in treating inflammation, cancer, allergies, diabetes, angiocardiopathy, and neurodegenerative diseases [4]. As seen in Figure 4, the contents of benzoylpaeoniflorin and benzoic acid occupied 65.66%, 68.18%, and 68.36% of the total polyphenol content after adsorption deacidification, alkali refining, and oil-hexane miscella deacidification, respectively.

The content of benzoic acid was significantly reduced after adsorption deacidification, alkali refining, and oil-hexane miscella deacidification (*p* < 0.05), from 10.92 mg/kg to 5.73 mg/kg, 6.83 mg/kg, and 6.92 mg/kg, respectively. While the content of benzoylpaeoniflorin and paeoniflorin was increased by 6.22 mg/kg, 2.95 mg/kg and 2.66 mg/kg, and that of paeoniflorin by 2.75 mg/kg, 2.11 mg/kg, and 2.18 mg/kg, respectively. The reason may be that benzoic acid can promote the synthesis of paeoniflorin and its derivatives during the refining process [39]. However, the total content still showed a downward trend, and the loss was the most serious in the deacidification process. Polyphenols were reduced by 29.36%, 41.41%, and 43.30% after adsorption deacidification, alkali refining, and oil-hexane miscella deacidification, respectively. The decrease during adsorption deacidification was much smaller than that during alkali refining. The reason may be that polyphenols are polar compounds that can be degraded by high temperature and alkali conditions, and can be washed away through the washing phase of deacidification [12].

### 4.6. Effect of Deacidification on the Antioxidant Capacities and Oxidative Stabilities of Oils

As can be seen in Table 2, the contents of TPC, DPPH, and FRAP were between 3.04~6.15 mg/kg, 8.53~16.16 μmoL TE/100 g, and 6.90~24.80 μmol TE/100 g oil, respectively. A significant decrease in the loss of antioxidant activity occurred during the acid neutralization step (*p* < 0.05). One possible explanation for these differences in the results of antioxidant activity between different deacidification modalities may be due to different mechanisms of deacidification. DPPH involves mainly the reaction of a rapid hydrogen atom transfer process from the phenate anion to the DPPH [40].

During deacidification, the abstraction of hydrogen atoms from phenolic compounds by DPPH becomes an edge reaction pathway due to occurring very slowly in strong hydrogen-bonding solvents such as methanol and ethanol. This results in the formation of phenolic acids and DPPH being reduced, and the reduction ability is weakened at the same time [41]. At 120 °C the OSI of PSO was only 0.10 h, which was lower than that of linseed oil and higher than walnut oil [42], mainly due to high UFA content and high acid value. After deacidification, the three processes could prolong the oxidation stabilization time, which increased to 2.52 h, 1.27 h, and 1.93 h, respectively. This increase was due to the acid value significantly decreasing. Among them, the OSI value of adsorption refining changed the most, and the stability was significantly enhanced. In addition to deacidification, it was also possible that the loss of bioactive components was not as great as that during the other two processes, thereby maximizing the retention of active components.

### 4.7. Analysis of PCA

**Figure 5:**
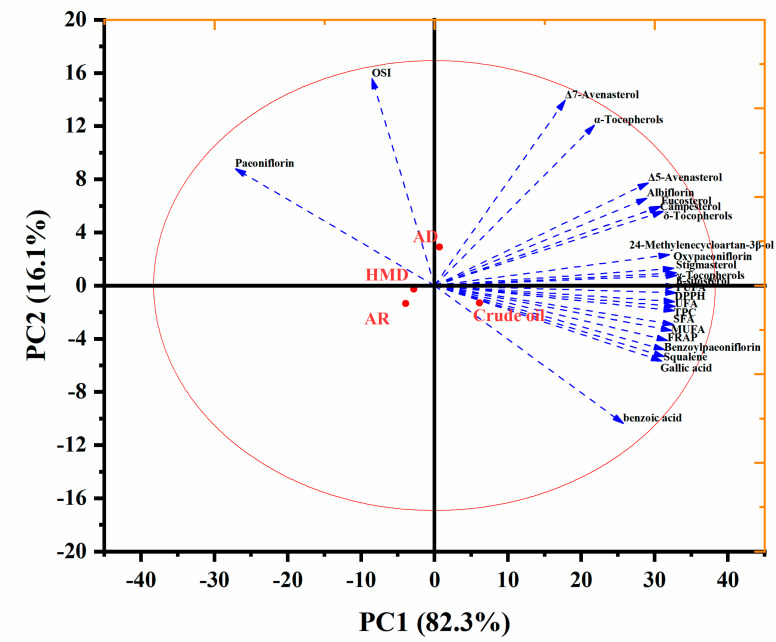
PCA of nutrient components in peony seed oil by deacidification.

The impact of different deacidification technologies on the bioactive compounds of PSO was studied using multivariate analysis. The results of PCA were presented in loading and score (Figure 5) plots.

The relative contribution of principal components (PC) was determined by only considering eigenvalues greater than one. Figure 5 shows variables that accounted for the total variability of 98.4% for PC1 (82.3%) and PC2 (16.1%) in the data set. Compared with deacidified oil, crude oil had higher fatty acid content and other bioactive content, such as benzoic acid, squalene, gallic acid, and FRAP, but it had lower paeoniflorin, OSI, α-tocopherol, and Δ-7 avenasterol content. At the same time, compared with the other two refining methods, it can be seen that the OSI and paeoniflorin content of PSO after adsorption increased, and the loss of active components was less. The values of the OSI and paeoniflorin content of PSO changed significantly after refining and were distributed on the left side of the figure. Chemical refining and mixed refining were also both distributed on the left side, which indicates that their fatty acid content and the biologically active ingredients were both greatly reduced, and they were all negatively correlated. Therefore, we divided the PSOs deacidification process research into three different groups in addition to the crude oil group. These include adsorption deacidification as a group, chemical adsorption as a group, and mixed refining as a group. Overall, except for OSI and paeoniflorin, the chemical composition and antioxidant activity of PSO decreased significantly after deacidification.

## 5. Conclusions

This study provided new findings regarding the effects of three deacidification processes on the quality of peony seed oil. In summary, adsorption deacidification (alkaline fiber addition of 3%, adsorption temperature of 50 °C, and adsorption time of 60 min,), alkali refining deacidification (NaOH addition of 18 Be, heating temperature of 60 °C and heating time of 10 min), and mixed deacidification (oil concentration of 50 g/100 g, heating temperature of 50 °C, and alkali addition of 12%) were compared, and the results showed that the deacidification rates of the three processes were 88.29%, 98.11% and 97.76%, respectively. Additionally, during the process optimization of the alkaline microcrystalline cellulose deacidification, it was found that alkaline microcrystalline cellulose is suitable for oils with an acid value of about 6 (KOH mg/g), which paves the way for an eco-friendly and sustainable material for the effective removal of FFA from the fiber material. During the study, the three processes had a significant impact on the composition of lipid companions, especially γ-tocopherol, which was reduced by 66.67 mg/kg, 132.78 mg/kg, and 121.95 mg/kg, respectively. After the three different deacidification processes, the total retention rates of sterols were 91.96%, 73.85%, and 76.45%, respectively, and deacidification significantly reduced the benzoic acid content by 18 to 27%. The three deacidification processes could extend the oxidative stabilization time from 1.07 h to 2.52 h, 1.27 h, and 1.93 h, respectively, which could extend the shelf life of PSO. This study contributes to future research on the adsorption deacidification treatment of PSO mass.

## Figures and Tables

**Table 1:** Fatty acid composition, tocopherols, and phytosterols contents in PSOs were obtained from crude oil, adsorption deacidification, alkali refining, and oil-hexane miscella deacidification.

	Crude Oil	AD	AR	HMD
**Fatty Acid (g/100 g)**				
C16:0	5.18 ± 0.20 ^c^	4.38 ± 0.00 ^ab^	4.09 ± 0.17 ^a^	4.12 ± 0.01 ^a^
C18:0	1.83 ± 0.034 ^c^	1.55 ± 0.01 ^b^	1.41 ± 0.04 ^a^	1.47 ± 0.08 ^ab^
C18:1	26.01 ± 1.07 ^c^	21.84 ± 0.01 ^ab^	20.28 ± 0.85 ^a^	21.04 ± 0.04 ^ab^
C18:2	29.16 ± 1.24 ^b^	24.36 ± 0.01 ^a^	22.63 ± 0.90 ^a^	23.11 ± 0.14 ^ab^
C18:3	42.97 ± 2.11 ^a^	41.87 ± 0.03 ^a^	39.05 ± 1.52 ^a^	40.57 ± 0.06 ^ab^
SFAs	7.01 ± 0.23 ^d^	5.93 ± 0.01 ^bc^	5.5 ± 0.21 ^a^	5.59 ± 0.06 ^ab^
MUFA	26.01 ± 1.07 ^c^	21.84 ± 0.01 ^ab^	20.29 ± 0.85 ^a^	21.04 ± 0.04 ^ab^
PUFA	72.13 ± 3.34 ^c^	66.22 ± 0.03 ^ab^	61.68 ± 2.42 ^a^	63.68 ± 0.19 ^ab^
UFAs	98.14 ± 4.41 ^c^	88.06 ± 0.05 ^ab^	81.96 ± 3.27 ^a^	84.72 ± 0.24 ^ab^
**Sterol (mg/kg)**				
Squalene	60.61 ± 0.70 ^c^	9.74 ± 0.86 ^b^	5.90 ± 0.17 ^a^	6.31 ± 0.33 ^a^
Campesterol	42.66 ± 0.84 ^d^	38.50 ± 0.33 ^c^	18.06 ± 0.27 ^a^	19.98 ± 0.19 ^b^
γ-Stigmasterol	27.53 ± 0.51 ^c^	21.41 ± 1.58 ^b^	15.08 ± 0.25 ^a^	15.91 ± 0.94 ^a^
β-Sitosterol	1684.62 ± 3.71 ^c^	1539.51 ± 11.86 ^b^	1422.18 ± 10.42 ^a^	1431.27 ± 27.90 ^a^
Fucosterol	772.53 ± 2.54 ^d^	718.99 ± 6.53 ^c^	473.11 ± 13.55 ^a^	533.76 ± 19.01 ^b^
Δ-5-Avenasterol	145.85 ± 2.23 ^b^	143.91 ± 7.76 ^b^	76.78 ± 3.11 ^a^	85.92 ± 5.38 ^a^
Δ-7-Avenasterol	22.61 ± 1.29 ^c^	28.71 ± 1.66 ^d^	15.30 ± 0.70 ^a^	19.68 ± 0.66 ^b^
24-Methylenecycloartan-3β-ol	253.63 ± 9.20 ^c^	211.64 ± 2.59 ^b^	151.80 ± 1.95 ^a^	141.88 ± 2.35 ^a^
Total	2949.43 ± 1.92 ^d^	2702.67 ± 7.63 c	2172.31 ± 30.25 ^a^	2248.38 ± 51.73 ^b^
**Tocopherols (mg/kg)**				
α-Tocopherols	47.88 ± 0.35 ^a^	48.17 ± 0.081 ^a^	47.14 ± 0.180 ^a^	47.59 ± 0.54 ^a^
γ-Tocopherols	843.12 ± 1.99 ^c^	776.45 ± 3.20 ^b^	590.34 ± 0.87 ^a^	621.16 ± 9.83 ^a^
σ-Tocopherols	7.12 ± 0.17 ^a^	7.64 ± 0.57 ^a^	7.85 ± 0.37 ^a^	7.05 ± 0.93 ^a^
Total	898.12 ± 2.52 ^d^	832.26 ± 3.85 ^c^	645.33 ± 1.43 ^a^	675.80 ± 10.22 ^b^

Values are means ± standard deviations, n = 3 replicates per treatment. Different superscript-case letters are significantly different at *p* ≤ 0.05 (one-way ANOVA and Duncan’s test, *p* ≤ 0.05). AD: adsorption deacidification, AR: alkali refining, HMD: oil-hexane miscella deacidification; SFAs, saturated fatty acids; MUFA: monounsaturated fatty acid; PUFA: polyunsaturated fatty acid; UFAs, unsaturated fatty acids.

**Table 2:** Effect of adsorption deacidification, alkali refining, oil-hexane miscella deacidification on the antioxidant capacities and oxidative stability index of PSO.

	TPC (mg GAE/100 g)	DPPH (μmoL TE/100 g)	FRAP (μmoL TE/100 g)	OSI (h)
Crude oil	6.15 ± 0.30 ^b^	16.16 ± 1.15 ^c^	24.80 ± 1.32 ^d^	1.07 ± 0.07 ^a^
AD	4.14 ± 0.13 ^a^	10.36 ± 0.33 ^b^	11.14 ± 1.11 ^b^	2.52 ± 0.06 ^c^
AR	3.14 ± 0.13 ^a^	6.46 ± 0.33 ^a^	6.90 ± 0.43 ^a^	1.27 ± 0.05 ^b^
HMD	3.04 ± 0.15 ^a^	8.54 ± 0.65 ^b^	10.17 ± 0.54 ^b^	1.93 ± 0.05 ^c^

Values are means ± standard deviations, n = 3 replicates per treatment. Different superscript-case letters are significantly different at *p* ≤ 0.05 (one-way ANOVA and Duncan’s test, *p* ≤ 0.05). AD: adsorption deacidification, AR: alkali refining, HMD: oil-hexane miscella deacidification; TPC: Total phenol content; DPPH: 2,2-diphenyl-1-picrylhydrazyl; FRAP: ferric-reducing antioxidant power; OSI: Oil Stability Index.

## Data Availability

Data is contained within the article.

## References

[1] Liu P., Zhang Y., Xu Y.-F., Zhu X.-Y., Xu X.-F., Chang S., Deng R.-X. (2018). Three new monoterpene glycosides from oil peony seed cake. Ind. Crop Prod..

[2] Liu P., Zhang L.N., Wang X.S., Gao J.Y., Yi J.P., Deng R.X. (2019). Characterization of Paeonia ostii seed and oil sourced from different cultivation areas in China. Ind. Crop Prod..

[3] Li S., Yuan R., Chen L., Wang L., Hao X.H., Wang L.J., Zheng X.C., Du H. (2015). Systematic qualitative and quantitative assessment of fatty acids in the seeds of 60 tree peony (Paeonia section Moutan DC.) cultivars by GC-MS. Food Chem..

[4] Wei X.-B., Xue J.Q., Wang S.-L., Xue Y.Q., Lin H., Shao X.F., Xu D.H., Zhang X.X. (2018). Fatty acid analysis in the seeds of 50 Paeonia ostii individuals from the same population. J. Intergr. Agr..

[5] Wang H., Li D.M., Wu Y., Ding Y. (2020). Preparation of tungstophosphoric acid intercalated MgAl layered double hydroxides with a tunable interlayer spacing and their catalytic esterification performance in the deacidification of model crude oil. J. Fuel Chem. Technol..

[6] Chang M., Wang Z., Zhang T., Wang T., Liu R., Wang Y., Jin Q., Wang X. (2020). Characterization of fatty acids, triacylglycerols, phytosterols and tocopherols in peony seed oil from five different major areas in China. Food Res. Int..

[7] Yang X., Zhang D., Song L.M., Xu Q., Li H., Xu H. (2017). Chemical Profile and Antioxidant Activity of the Oil from Peony Seeds (Paeonia suffruticosa Andr.). Oxid. Med. Cell Longev..

[8] Su J., Wang H., Ma C., Lou Z., Liu C., Tanvir Rahman M., Gao C., Nie R. (2015). Anti-diabetic activity of peony seed oil, a new resource food in STZ-induced diabetic mice. Food Funct..

[9] Wang Z., Zheng C., Huang F., Liu C., Huang Y., Wang W. (2021). Effects of Radio Frequency Pretreatment on Quality of Tree Peony Seed Oils: Process Optimization and Comparison with Microwave and Roasting. Foods.

[10] Adhami K., Asadollahzadeh H., Ghazizadeh M. (2019). A novel process for simultaneous degumming and deacidification of Soybean, Canola, and Sunflower oils by tetra butyl phosphonium phosphate ionic liquid. J. Ind. Eng. Chem..

[11] Wang X., Wang X., Xie D. (2021). A novel method for oil deacidification: Chemical amidation with ethanolamine catalyzed by calcium oxide. LWT-Food Sci. Tenhnol..

[12] Chew S.C., Ali M.A. (2021). Recent advances in ultrasound technology applications of vegetable oil refining. Trends Food Sci. Tech..

[13] Chew S.-C., Tan C.-P., Nyam K.-L. (2017). Optimization of neutralization parameters in chemical refining of kenaf seed oil by response surface methodology. Ind. Crop Prod..

[14] Pan F., Wen B., Wang X., Ma X., Zhao J., Liu C., Xu Y., Dang W. (2019). Effect of the chemical refining process on perilla seed oil composition and oxidative stability. J. Food Process Pres..

[15] Wang X., Li C., Contreras M.d.M., Verardo V., Gómez-Caravaca A.M., Xing C. (2020). Integrated Profiling of Fatty Acids, Sterols and Phenolic Compounds in Tree and Herbaceous Peony Seed Oils: Marker Screening for New Resources of Vegetable Oil. Foods.

[16] Shi L., Zheng L., Zhao C., Huang J., Jin Q., Wang X. (2018). Effects of deacidification methods on high free fatty acid containing oils obtained from sea buckth (*Hippophaë rhamnoides* L.) ornberry. Ind. Crop Prod..

[17] Gonçalves C.B., Rodrigues C.E.C., Marcon E.C., Meirelles A.J.A. (2016). Deacidification of palm oil by solvent extraction. Sep. Purif. Technol..

[18] Wang B., Sun S., Yao N., Chu C. (2021). A novel method for simultaneous degumming and deacidification of corn oil by miscella refining in one step. LWT-Food Sci. Technol..

[19] Ferreira M.C., Toledo Hijo A.A.C., Farias F.O., Batista E.A.C., Maximo G.J., Meirelles A.J.A. (2022). In search of sustainable alternatives for vegetable oils deacidification using the oligomerice-based ionic liquid approach. Fluid Phase Equilibr..

[20] Wang X., Lu J., Liu H., Jin Q., Wang X. (2016). Improved deacidification of high-acid rice bran oil by enzymatic esterification with phytosterol. Process Biochem..

[21] Xu Q., Lan D., Liu X., Yang B., Sun-Waterhouse D., Liao S., Wang W., Wang Y. (2021). Enzymatic deacidification of alpha-linolenic acid -enriched oils with negligible change in triacylglycerol composition. Process Biochem..

[22] Bao Y., Zhou Q., Zhang M., Zhang H., Luan Q., Zhou W., Tang H., Huang F. (2019). Wet-spun nanoTiO2/chitosan nanocomposite fibers as an efficient and retrievable absorbent for the removal of free fatty acids from edible oil. Carbohydr. Polym..

[23] Feng K., Huang Z., Peng B., Dai W., Li Y., Zhu X., Chen Y., Tong X., Lan Y., Cao Y. (2020). Immobilization of Aspergillus niger lipase onto a novel macroporous acrylic resin: Stable and recyclable biocatalysis for deacidification of high-acid soy sauce residue oil. Bioresour. Technol..

[24] Rezaei M., Khaledabad M.A., Kia E.M., Ghasempour Z. (2020). Optimization of grape juice deacidification using a mixture of adsorbents: A case study of Pekmez. Food Sci. Nutr..

[25] Devi L.S., Das A.J., Das A.B. (2022). Characterization of high amylose starch-microcrystalline cellulos floatable gel for enhanced gastrointestinal retention and drug delivery. Carbohydr. Polym. Technol. Appl..

[26] Garba Z.N., Lawan I., Zhou W., Zhang M., Wang L., Yuan Z. (2020). Microcrystalline cellulose (MCC) based materials as emerging adsorbents for the removal of dyes and heavy metals—A review. Sci. Total Environ..

[27] Chen S.Q., Meldrum O.W., Liao Q., Li Z., Cao X., Guo L., Zhang S., Zhu J., Li L. (2021). The influence of alkaline treatment on the mechanical and structural properties of bacterial cellulose. Carbohydr. Polym..

[28] Mubarak M.F., Zayed A.M., Ahmed H.A. (2022). Activated Carbon/Carborundum@Microcrystalline Cellulose core-shell nano-composite: Synthesis, characterization, and application for heavy metals adsorption from aqueous solutions. Ind. Crop Prod..

[29] National Health Commission (2016). National Standard of the People’s Republic of China.

[30] Cong Y., Cheong L.Z., Huang F., Zheng C., Wan C., Zheng M. (2019). Effects of microwave irradiation on the distribution of sinapic acid and its derivatives in rapeseed and the antioxidant evaluation. LWT-Food Sci. Technol..

[31] Azadmard-Damirchi S., Habibi-Nodeh F., Hesari J., Nemati M., Achachlouei B.F. (2010). Effect of pretreatment with microwaves on oxidative stability and nutraceuticals content of oil from rapeseed. Food Chem..

[32] Sun W., Cai C., Duan Miao L., Ma Z., Chen S. (2020). Preparation of Oil Deacidification Agent Using Microcrystalline Cellulose and Its Application in Adsorption of Free Fatty Acids from Oil. Food Indust..

[33] Lu J., Wen J., Zhang Y., Shu Y. (2020). Application of Alkaline Microcrystalline Cellulose in Adsorption of Free Fatty Acids from Rapeseed Oil. Food Indust..

[34] Hu C.C., Ji Y.T. (2008). The stability of alkaline sorbent and adsorption balance in oil deacidification. Cereal Oil.

[35] Wang W., Liu C., Huang F., Li W., Yang B. (2016). Application of alkaline microcrystalline cellulose in deacidfication of tea seed oil. Chin. J. Oil Crop Sci..

[36] Gebauer S.K., Psota T.L., Harris W.S., Kris-Etherton P.M. (2006). n-3 fatty acid dietary recommendations and food sources to achieve essentiality and cardiovascular benefits. Am. J. Clin. Nutr..

[37] Hu F.B. (2001). The balance between ω-6 and ω-3 fatty acids and the risk of coronary heart disease. Nutrition.

[38] Zheng L., Jin J., Shi L., Huang J., Chang M., Wang X., Zhang H., Jin Q. (2020). Gamma tocopherol, its dimmers, and quinones: Past and future trends. Crit. Rev. Food Sci..

[39] Bai Z.Z., Ni J., Tang J.M., Sun D.Y., Yan Z.G., Zhang J., Niu L.X., Zhang Y.L. (2021). Bioactive components, antioxidant and antimicrobial activities of Paeonia rockii fruit during development. Food Chem..

[40] Ballus C.A., Meinhart A.D., de Souza Campos F.A., Godoy H.T. (2015). Total Phenolics of Virgin Olive Oils Highly Correlate with the Hydrogen Atom Transfer Mechanism of Antioxidant Capacity. J. Am. Oil. Chem. Soc..

[41] Liu R., Liu R., Shi L., Zhang Z., Zhang T., Lu M., Chang M., Jin Q., Wang X. (2019). Effect of refining process on physicochemical parameters, chemical compositions and in vitro antioxidant activities of rice bran oil. LWT-Food Sci. Technol..

[42] Ma Y., Jiang Q., Cheng H., Luo J., Li Y., Zhang H. (2022). Composition and Oxidative Stability of Fatty Acids in Ten Small Trade Vegetable Oils. Food Sci. Biotechnol..

